# Mesenchymal Stem Cells-derived Exosomes: A New Possible Therapeutic Strategy for Parkinson’s Disease?

**DOI:** 10.3390/cells8020118

**Published:** 2019-02-02

**Authors:** Helena Vilaça-Faria, António J. Salgado, Fábio G. Teixeira

**Affiliations:** 1 Life and Health Sciences Research Institute (ICVS), School of Medicine, University of Minho, 4710-057 Braga, Portugal; helena.mv.faria@gmail.com; 2 ICVS/3B’s Associate Lab, PT Government Associated Lab, Braga/Guimarães, Portugal

**Keywords:** mesenchymal stem cells, secretome, exosomes, Parkinson’s disease, microRNAs

## Abstract

Parkinson’s disease (PD) is the second most prevalent neurodegenerative disorder worldwide. Clinically, it is characterized by severe motor complications caused by a progressive degeneration of dopaminergic neurons (DAn) and dopamine loss. Current treatment is focused on mitigating the symptoms through administration of levodopa, rather than on preventing DAn damage. Therefore, the use and development of neuroprotective/disease-modifying strategies is an absolute need, which can lead to promising gains on PD translational research. Mesenchymal stem cells (MSCs)–derived exosomes have been proposed as a promising therapeutic tool, since it has been demonstrated that they can act as biological nanoparticles with beneficial effects in different pathological conditions, including PD. Thus, considering their potential protective action in lesioned sites, MSCs-derived exosomes might also be active modulators of the neuroregeneration processes, opening a door for their future use as therapeutical strategies in human clinical trials. Therefore, in this review, we analyze the current understanding of MSCs-derived exosomes as a new possible therapeutic strategy for PD, by providing an overview about the potential role of miRNAs in the cellular and molecular basis of PD.

## 1. Introduction

Described by James Parkinson in 1817, Parkinson’s disease (PD) is the second most common chronic neurodegenerative disease in the world, affecting over 10 million people, and approximately 1% of the world population over 60 years old [1]. Pathologically, PD is characterized by the degeneration of dopaminergic neurons (DAn) and by the deficiency of dopamine production in several dopaminergic networks. The loss of dopaminergic neurons is also linked with the formation/accumulation of Lewy bodies (LB; protein aggregates of α-synuclein) in the intraneuronal structure, affecting the normal functioning of those cells. From the networks impaired, the most affected one is the nigrostriatal pathway at the level of the substantia nigra pars compacta (SNpc) and the striatum (STR) [2], initially with an asymmetric onset that becomes bilateral as the disease progresses [3]. However, there are other brain areas presenting the above referred hallmarks, such as the olfactory bulb, neocortex, limbic system, and brainstem cells nuclei, suggesting a prion disease-like propagation and progression [4]. With this insight, a model was proposed, supporting LB transmission among cells as a possible route for disease onset and progression. This model, called the Braak system, is divided in several stages, in which the autonomic nervous system (ANS) is the first affected by the pathology (stage 0), followed by the dorsal motor nucleus of the vagus (DMV) and the anterior olfactory nucleus (stage 1), spreading to the locus coeruleus (LC), SNpc, and basal forebrain (stage 2) and finally, to the neocortex, hippocampus, and basal ganglia (final stages) [5]. As a result, when DAn death exceeds a threshold in the nigrostriatal pathway it affects the patients’ motor system. Therefore, PD is clinically recognized by a core of motor symptoms, including bradykinesia, rigidity, tremor, and postural instability, which are used in the establishment of its diagnosis [6]. However, non-motor symptoms, such as depression, sleep disorders, dementia, and peripheral impairments, have also been linked with functional disabilities, preceding the appearance of the motor symptomatology [7]. Thus, the development of management strategies is crucial, in which the diagnosis and the evaluation of the condition of the patient should be accurate, being followed by the development and application of personalized strategies, aiming to ameliorate the patient’s quality of life [8].

## 2. Molecular and Cellular Aspects of Parkinson’s Disease

As already mentioned, the major pathological feature of PD is the progressive loss of DAn in the nigrostriatal system due to the presence of intraneuronal inclusions, namely LB [3]. Along with SNpc’ DAn, other neural populations of the central (CNS) and peripheral nervous systems (PNS) are affected by PD pathophysiology. For instance, in the PNS, the most affected subdivision is the ANS, in which norepinephrine (NE) neurons innervating the heart and skin [9,10], as well as DAn of the enteric nervous system (ENS) [11], are lost in PD. Actually, it is believed that the loss of these enteric DAn leads to orthostatic hypotension, hyperhidrosis, and constipation, some of the less known symptoms correlated with PD development. Regarding the CNS, almost all PD patients lose its neuromelanin positive-catecholamine DAn at the levels of the SNpc and LC, something that is also observed in DMV [12]. Still, DAn from the ventral tegmental area (VTA), retrorubal field (RRF), raphe nuclei (RN), and basal nucleus of Meynert (BNM) are also lost in PD, but to a lesser extent [13]. Notwithstanding, although several brain regions are claimed as being affected by PD pathophysiology, only the selective loss of the SNpc’ DAn recognize the core symptoms of PD. Indeed, SNpc’ DAn are one of the longest and most densely arborated neurons of the brain, projecting to the STR through a longer and thinner unmyelinated axon [14]. In addition, studies have also suggest that as DAn axons make an elevated number of synaptic connections, they appear to be more prone to damage [15], as it has been indicated that the risk of local α-synuclein misfolding increases [16]. Furthermore, studies have also suggested that SNpc DAn present a pacemaker activity that is regulated by specific Ca^2+^ channels, leading to an increase in the cytosolic Ca^2+^ concentration [17]. Such an increase has been correlated with the occurrence of cellular stress, leading to the formation of reactive oxygen species (ROS), which are known to be detrimental to DAn viability [18]. The mitochondria is responsible for the DAn calcium homeostasis, which in turn increases energy demand, contributing to the vulnerability of these neurons [19]. In addition to this, dopamine itself could also be detrimental to DAn viability, as studies have demonstrated that the increase of free cytosolic dopamine caused by an unbalanced homeostasis at several levels (synthesis, storage, degradation, and/or distribution in the synaptic vesicles) favors ROS production and oxidative stress, leading to DAn damage [20,21]. Moreover, the SNpc’ DAn present a dark colored pigment, called neuromelanin (NM), which acts as a reservoir of iron, metals, and other toxic substances, having a neuroprotective effect [22]. In addition to neuroprotection, NM has recently been proposed as a promising biomarker for PD [23]. However, (DAn) dying neurons release NM to the extracellular space, creating deposits that induce microglial activation, chemotaxis, and proliferation, thus supporting SNpc inflammation and neuronal degeneration [24]. These multifactorial features led to the study of the underlying mechanisms responsible for the loss of DAn linked to PD.

Rationally, the first question to be answered is how does PD begin at the cellular level? Although this answer remains under discussion, several studies have demonstrated that the degeneration in PD initiates in the synaptic and axonal terminals, beginning in the STR, and in a retrograde manner, progresses to the SNpc’ DAn somas [25]. In fact, the literature shows that, at the time of the motor symptoms onset, 30% of the SNpc’ DAn are lost, while 50–60% of the axon terminals in the STR are already degenerated [26]. However, the exact mechanisms of such degeneration is still not understood and some concepts have been proposed throughout time. The most relevant mechanisms involving PD include the disruption of protein clearance pathways, the accumulation of α-synuclein protein aggregates, mitochondrial dysfunction, glutamate/calcium excitotoxicity, oxidative stress, neuroinflammation, and genetic mutations [27]. Most of these mechanisms are related to DAn sensitivity and susceptibility to degeneration, as previously described. However, cell death may be caused by specific genetic mutations, which in turn affect several PD interlayers. Pathogenic mutations in PD can lead to protein degradation systems’ (ubiquitin-proteasome and autophagy-lysosome system) failure, which leads to the accumulation of misfolded α-synuclein, defective mitochondria, thereby creating intercellular oxidative stress, and thus leading to DAn degeneration [28,29]. Although it represents less than 10% of all PD cases, at least 17 autosomal dominant and autosomal recessive gene mutations, namely, α-SYN (SNCA), PARKIN (PRKN), ubiquitin C-terminal hydrolase L1 (UCHL-1), PTEN-induced putative kinase 1 (PINK1), protein deglycase (DJ-1, PARK7), and leucine-rich repeat kinase 2 (LRRK2, PARK8) genes, among others have been identified [27]. Notwithstanding, although most of the PD cases are sporadic (idiopathic), being caused by an interaction between genetic and environmental factors [30], such as aging, inflammation, and exposure to neurotoxic agents (e.g., pesticides, such as rotenone and paraquat), both sporadic and familial forms of PD have mutual molecular pathways, as shown in Table 1, making PD a multi-targeted disease in which new strategies, with a multimodal action, may be of particular value [31]. 

## 3. Parkinson’s Disease Treatments: Do We Have What Is Needed?

The loss of DAn and reduced dopamine production underlies the reasoning of the PD gold standard treatment, which is still the administration of levodopa [32,33]. However, this strategy remains insufficient to recover lost DAn, or to avoid PD progression, as its extended use, associated with the needs of increased dosages, is linked with secondary effects, such as motor fluctuations and behavioral changes (e.g., impulsivity and addiction) [34]. The field’s current view is that combinatory strategies may overcome the limitations of single levodopa administration, particularly by combining the latter with other PD pharmacological treatments. Such combined treatments have demonstrated the ability to enhance and prolong levodopa efficacy by involving the use dopamine receptor agonists (e.g., ropinirole, pramipexole, piribedil) [35]; inhibitors of peripheral enzymes, such as levodopa decarboxylase (e.g., carbidopa and benserazide) [36] or catechol-*O*-methyl transferase (COMT) (e.g., entacapone, tolcapone, and, more recently, opicapone) [37,38]; and inhibitors of central enzymes, such as monoamine-oxidase B (MAO-B) (e.g., selegiline, rasagiline, or safinamide) [8,39] for oral intake. Besides these, throughout the years, other options were developed without the direct application of levodopa. This includes other dopamine agonists, such as rotigotine by transdermal application [40], and apomorphine by subcutaneous administration [41]. Also, then *N*-methyl-D-aspartate (NMDA) receptor antagonist (e.g., amantadine) was found to improve PD motor impairments, by reducing dyskinesia and other PD-related complications [42]. Surgical procedures, such as deep brain stimulation (DBS), have also been used in the treatment of PD, being a procedure that comprises the delivery of electrical pulses to neurons through a neurostimulator implantation, either in the subthalamic nucleus or in the internal part of the globus pallidus, leading to symptomatic relief [43]. 

In addition to these pharmacological and surgical treatments, in the last years, a large number of new approaches have been developed to verify the effect of molecular agents (e.g., adenosine receptor antagonists, anti-apoptotic agents, and antioxidants) and non-pharmacotherapies (e.g., viral vector gene therapy, microRNAs, transglutaminases, and RTP801) in the treatment of PD [44]. However, although promising results have been experimentally and clinically obtained with several drugs and surgical experiments, yet the challenge remains to show a clinical proof of arrest of delay of DAn loss in PD [8]. Therefore, there is an urgent need for the establishment of innovative therapies that adequately target PD, particularly by inducing neuroprotection of the surviving DAn within the SNpc-STR pathway, as well as stimulating the differentiation of new ones, so that the dopamine balance can be re-established. With the advent of stem cell biotechnology, new routes are currently being explored, particularly those aiming to protect DAn, as it is the case of human mesenchymal stem cells (MSCs)-derived exosomes [45,46]. Therefore, in the scope of this review, we will discuss the current understanding of MSCs-derived exosomes by reviewing recent experimental data addressing the therapeutical potential of those vesicles in the context of PD.

## 4. Mesenchymal Stem Cells (MSCs)-Derived Exosomes and Parkinson’s Disease

### MSCs-Derived Exosomes 

As we have previously reviewed, according to the definition introduced by the International Society for Cellular Therapy (ISCT), there are some minimal criteria for the identification of MSCs populations, namely (1) the adherence to plastic in standard culture conditions; (2) the positive expression of specific markers, like CD73, CD90, and CD105, and negative expression of hematopoietic markers, like CD34, CD45, HLA-DR, and CD14, or CD11B, CD79α, or CD19; and (3) in vitro differentiation into at least osteoblasts, adipocytes, and chondroblasts [47,48]. Therefore, MSCs are a multipotent non-hematopoietic stem cell population that has emerged in the last decade as a promising therapeutic tool for the treatment of several disorders, including PD [45,47]. This potential is associated with their widespread availability throughout the human body, namely in the bone marrow, adipose tissue, brain, dental pulp, placenta, umbilical cord blood, and Wharton’s jelly [47,49]. Notwithstanding, it is important to highlight that although all these populations are within the definition of MSCs, they can have subtle differences, mainly in their membrane antigen markers [47]. Indeed, studies have demonstrated that such differences may be the result of different cell culture protocols in their isolation and expansion or, alternatively, be related with the tissue source from which they are being isolated [50,51]. Although, from the application point of view, studies have shown that after (intracranial) transplantation, these cells act as promoters of immunomodulation, neuroprotection, and neuronal differentiation [52,53]. These effects are essentially mediated by the products that are released by MSCs into the extracellular milieu, commonly defined as secretome [54]. MSC-secretome has been described as a complex mixture of soluble products composed by a proteic soluble fraction (constituted by growth factors and cytokines), and a vesicular fraction composed by microvesicles and exosomes, which are involved in the transference of proteins and genetic material (e.g., miRNA) to other cells, with promising therapeutic effects [45,47].

Our lab has shown that MSC-secretome acts as an important promoter of neuroprotection, neurodifferentiation, by modulating neural stem cells, neurons and glial cells, and axonal growth in vitro and in vivo environments [52,55,56,57,58,59,60,61]. More recently, we have revealed that the use of dynamic culturing conditions (through computer-controlled bioreactors) can further modulate MSC-secretome, generating a more potent neurotrophic factor cocktail [62,63]. In the context of PD, we have recently shown that its administration in the SNpc-STR pathway was able to partially revert the motor and histological symptoms of a 6-OHDA PD rat model [64], indicating that MSC-secretome can be used as a therapy for PD. Following on this work we have identified the presence of important neuroregulatory molecules in the secretome of MSCs, including BDNF, IGF-1, VEGF, Pigment epithelium-derived factor (PEDF), DJ-1, and Cystatin-C (Cys-C), that are being described as potential therapeutic mediators against PD [62,65], as well as matrix metalloproteinases (MMPs), namely MMP 2, known for being able to degrade alpha synuclein aggregates [65,66], and have correlated their presence with the impact observed in our in vitro and in vivo models.

In addition to this protein fraction, the secretome also presents a vesicular portion, which is composed by extracellular vesicles (EVs). The latter are important in cell-to-cell communication, as they are involved in the transference of proteins and genetic material to neighboring cells [67]. EVs are secreted by different cell types, such as neurons, microglia, epithelial, endothelial, and hematopoietic cells, and stem cells as MSCs [68]. According to the International Society for Extracellular Vesicles (ISEV), EVs are characterized by three minimal criteria: (1) Isolation from conditioned cell culture medium or body fluids, with negligible cell disruption; (2) quantification of one protein (at least) from three distinctive categories in the EV preparation-cytosolic proteins, transmembrane or lipid bound extracellular proteins, and intracellular proteins; and (3) vesicles characterization using at least two different technologies—by imaging (e.g., electron microscopy or atomic force microscopy) and EVs size distribution measurements (e.g., nanoparticle-tracking analysis or resistive pulse sensing) [69]. EVs are classified as microvesicles, exosomes, and apoptotic cell bodies [70] based on their size, origin, and cargo. Regarding their size, exosomes are the smallest type, being classified as vesicles with a range of 30-150 nm, while microvesicles and apoptotic bodies have a 50–1000 nm and 50–2000 nm diameter, respectively [71]. EVs are distinguished as exosomes if formed inside multivesicular bodies (MVBs) at the endolysosomal pathway and secreted upon MVBs fusion with the membrane, in contrast to microvesicles, which form from the sprouting of the plasma membrane, while apoptotic bodies originate from dying cells fragments [72]. Exosomes are the best characterized EV population and were first discovered in 1983 in maturing sheep retilocytes [73]. Exosomes present a phospholipid layer characterized by sphingolipids, ceramides, tetraspanins (CD63, CD9, CD81), fusion proteins (flotillins, CD9, annexin), integrins, heat shock proteins (HSC70 and HSC90), membrane transporters (GTPases), lysosomal proteins (Lamp2b), tumor sensitive gene (TSG101), and Alix [74]. Regarding their cargo, exosomes contain a variety of biomolecules, such as cell-type specific proteins, signaling peptides, lipids, and genetic material (e.g., miRNA, small RNA, genomic DNA, mRNA, long non-coding RNA, tRNA, cDNA, and mtDNA), which once released to the extracellular environment, are taken up by other cells [75]. This interaction can lead to changes in the cell phenotype or to a modulation of the cell activity, raising the question of whether exosomes can represent the basis for the creation of new therapeutical strategies under the (CNS) regenerative medicine field. Indeed, studies have remarkably explored and demonstrated exosomes as a delivery system of therapeutical signals or drugs due to their low immunogenicity, ability to cross the blood-brain barrier (BBB), and long half-life in circulation [76]. As described, different cell types secrete exosomes, however, in this review, we highlight the ones derived from the secretome of MSCs, since they show promising effects by triggering regenerative responses in different pathological conditions. MSC-derived exosomes were firstly isolated and described in 2010 from human MSCs-derived from embryonic stem cells (ESC) [77]. Actually, since their discovery, an increasing number of studies explored their regenerative potential using diverse in vitro and in vivo models of several pathological conditions by demonstrating that the uptake of MSCs-derived exosomes are able to stimulate angiogenesis and myogenesis, promote functional and morphologic rescue due to a decrease of oxidative stress and suppression of apoptosis, as well as the modulation of inflammatory responses [78,79,80,81,82,83].

Concerning CNS pathologies, MSCs-derived exosomes have also shown therapeutical benefits. For instance, in stroke, intravenous administration of MSCs-derived exosomes induced an increase of neurogenesis, neurite remodeling, and angiogenesis, facts that were correlated with a substantial improvement of animals’ functional recovery [84]. Such a tendency was also observed in a traumatic brain injury model, showing an inflammation reduction and good outcomes after MSCs-derived exosomes’ administration [85]. The injection of MSCs-derived exosomes has also been shown to be a possible treatment for spinal cord injury (SCI), by reducing inflammation and by promoting neuro-regeneration in rats after injury [86,87]. In neurodegenerative diseases, such as Alzheimer’s, studies have shown MSCs-derived exosomes expressing high levels of the amyloid β-degrading enzyme, neprilysin (NEP), leading to a decrease of brain Aβ levels [88], and thus having an impact on the disease progression. In the context of PD, MSCs-derived exosomes were found to rescue DAn in in vitro (6-OHDA) models of PD, providing a potential regenerative treatment for this disorder [89]. 

However, although promising results have been claimed by MSCs-derived exosomes, studies have also claimed that the exosomes content depends on the tissues where MSCs are originally isolated and the environment in which they are present, setting the need to further study the different functional exosomal properties. Such an assumption is in line with previous results published by our group, which demonstrated that MSCs from different sources have different secretome profiles, thereby indicating that such a difference in their secretion pattern may indicate that their secretome or derived vesicles may be specific to a condition of the CNS [65].

## 5. Exosomal Genetic Material Content: Are miRNAs Important in the Modulation of the Molecular and Cellular Issues of PD?

**Figure 1:**
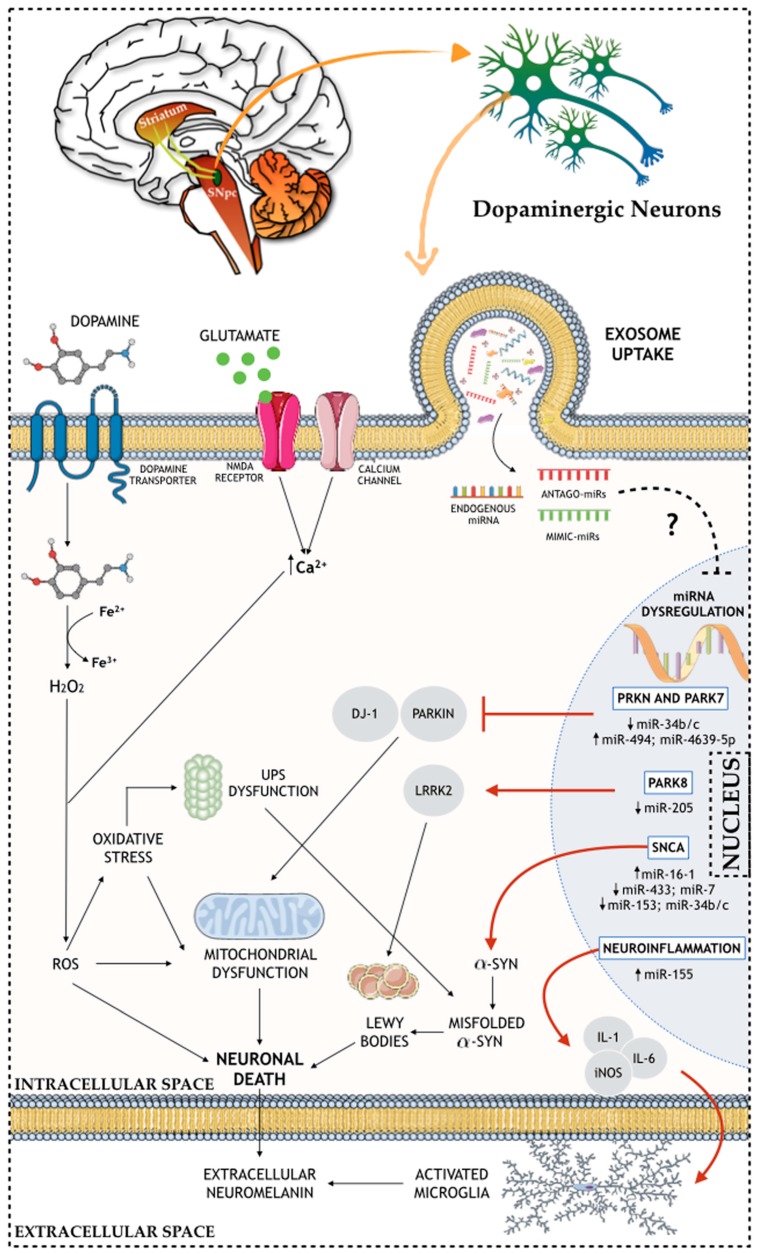
Schematic representation of the role of miRNAs in the molecular and cellular (e.g. nuclear, intracellular, and extracellular) mechanisms of PD brain.

As previously mentioned, one of the most common content of exosomes is the presence of genetic material, such as microRNAs (miRNAs) [90]. Actually, it has been indicated that numerous diseases, including PD, exhibit intense dysregulation of gene expression, specifically at the miRNA level [91]—Figure 1. In addition to its involvement in PD pathophysiology, exosome-derived microRNAs have also been identified as a potential tool for diagnosis biomarkers and targeted therapies.

miRNAs are the most studied class of non-coding RNAs (ncRNA), with between 21–25 nt, and are responsible for the regulation of specific genes through RNA messenger (mRNA) degradation or inhibition of their translation [92]. Still, miRNAs bind to the untranslated region (UTR) of the mRNA target and recruit the RNA induced silencing complex (RISC) in order to inhibit the expression of these targets, therefore, regulating specific gene expression, and presenting key roles in normal cellular physiology [93]. In animals, miRNAs are produced in two stages, starting from primary miRNAs (pri-miRNAs), and by the action of Drosha/DGCR8 RNase in the nucleus, and Dicer RNase in the cell cytoplasm [94]. This miRNA biogenesis pathway is of great importance and is essential for normal development since Dicer knockout (KO) mice are not able to survive beyond the embryonic stage [95]. Also, it was shown that impairments in Dicer in mice midbrain leads to a progressive loss of DAn [96], and post-mortem brain analysis showed DAn loss combined with LB, when the DGCR8 gene was deleted (chromosome 22q11.2 deletion syndrome) [97]. 

Several miRNA-mediated dysfunction networks in PD-related genes have recently been reported. Concerning the SNCA gene, several miRNAs have been suggested as α-synuclein modulators. For instance, interference in the binding between miR-433 and fibroblast growth factor 20 (FGF20) mRNA leads to increased levels of FGF20, which in turn also increases the levels of the α-synuclein protein in the cell [98]. Moreover, an abnormal increase of the miR-16-1 levels inhibits to a greater extent the translation of the HSP70 mRNA (protein that inhibits α-synuclein), which in turn also leads to an increase of the α-synuclein protein levels [99]. Also, PD-related pathogenic processes blocking miR-7, miR-153, and miR-34b/c from binding on their α-synuclein mRNA target automatically leads to increased levels of α-synuclein [100,101,102]. Regarding PRKN and PARK7 genes, they express, respectively, the PARKIN and DJ-1 proteins, which present important roles in the normal cell functioning and PD. PARKIN protein partakes in the proteasome-mediated degradation, and it is expressed in the mitochondria, where it binds to mtDNA, protecting it against damage promoted by oxidative stress conditions [103]. DJ-1 protein is considered an oxidative detector and it binds to PARKIN protein in oxidative stress conditions, protecting the mitochondria from oxidative stress [104]. Also, mutations in the PARK7 gene make DAn more susceptible to ROS-mediated damage [105]. In PD, a correlation was found between the decrease of miR-34b/c levels and the consequent decrease of the PARKIN and DJ-1 proteins in several brain areas [106]. Also, an upregulation of miR-494 and miR-4639-5p causes a direct reduction of DJ-1 protein expression, making DAn more vulnerable and prone to the PD phenotype [107,108]. Moreover, LRRK2 gene (PARK8) mutations cause sporadic PD associated to a neuropathology characterized by SNpc’ DAn loss, which is, in some cases, accompanied by the formation and presence of LB [109]. In fact, studies verified an increase of LRRK2 expression in PD patients when compared with controls, correlating this increase with a downregulation of miR-205 [110]. Another miRNA associated with the dopaminergic phenotype in PD is miR-133b, which is found to be downregulated in PD patients, and it regulates the transcriptional activator, Pitx3, an important factor in DAn development [111]. Additionally, other miRNAs were found to regulate the expression of genes involved in neuroinflammation, an important hallmark of PD. In this context, studies have found that miR-155 plays a key role in the upregulation of the inflammatory response to α-synuclein fibrils. This occurs by the fact that miR-155 is a modulator of proinflammatory molecules, such as IL-1, IL-6, TNF-α, and iNOS, leading to its upregulation [112]. Also, an miR-155 KO mice model showed that the lack of this miRNA prevented reactive microgliosis, as well as the loss of DAn triggered by the overexpression of α-synuclein [113]. In the same line of thought, miR-7, which was previously reported as an important factor in the regulation of α-synuclein levels, has also been presented as an important player in the modulation of neuroinflammation. For instance, the injection of miR-7 in the STR of an MPTP mouse model of PD was found to block NLRP3 inflammasome activation, leading to a remarkable attenuation of DAn death [114].

**Figure 2:**
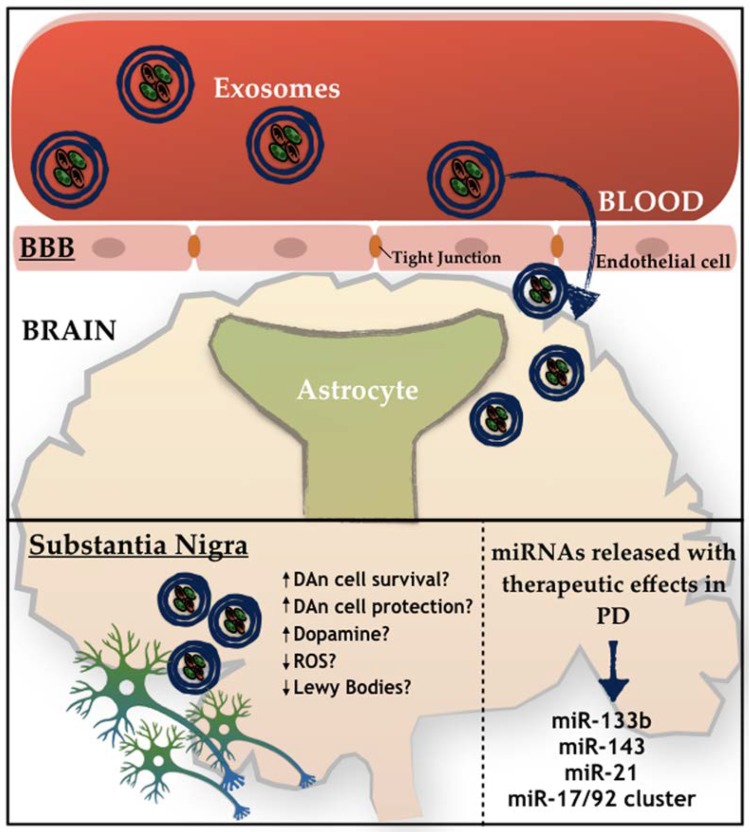
Schematic representation of the active role of exosomes on PD. How exosomes recognize and internalize other cells remains under discussion. Free-floating, adhesion, and antigen recognition have been described as mechanisms of cellular recognition, while soluble and juxtacrine signaling, fusion, phagocytosis, micropinocytosis, and receptor- and raft-mediated endocytosis have been described as mechanisms of exosomal internalization, as described by [75].

In addition to this involvement in PD pathophysiology, miRNAs are also being investigated as a potential source of PD biomarkers, in which the exosomes are being identified as a great use for diagnosis and prognosis of the disease. Indeed, Vizoso and colleagues [115] have recently proposed that MSCs-derived secretome is sufficient to significantly improve multiple biomarkers of the pathophysiology, making it a potential strategy to be used for the establishment and identification of promising PD biomarkers. As we have previously described, MSCs are able to secrete large quantities of exosomes carrying miRNAs, and such miRNAs may function not only as a novel class of promising biomarkers, but as modulators of multiple systems that could play critical roles in several diseases, including PD. Therefore, the possibility of using it as a potential therapeutic strategy for the treatment of PD is starting to emerge. To target the brain areas affected in PD, miRNAs must be delivered into the brain through a transport system able to cross the BBB—Figure 2. Due to the multi-faceted nature of exosomes, its application in clinics is something that could be envisaged in the near future [116]. However, firstly, some challenges need to be addressed, namely: (1) The correct (MSC) cell line; (2) exploration of the most efficient and reliable yield isolation technique associated to an efficient scalable production; (3) development of robust loading methods without damage to the exosomal integrity, in order to ensure an improved insight into PD cellular and molecular mechanisms, and finally to (4) address and plan possible strategies to improve (MSCs) exosomes’ targeting capability.

MSCs-derived exosomes may constitute a new key solution. Indeed, several studies show that MSCs-derived exosomes are able to transfer miRNAs to neuronal cells, in which exosomes enriched in miR-133b can promote neurite outgrowth [117], which is of great benefit for PD, as it is one of the miRNAs that is normally downregulated in the disease. Still, miR-143 and miR-21 were also found to be present in MSCs-derived exosomes, being described as important players in immune response modulation and in neuronal death associated with an environment of chronic inflammation [118]. Similarly, an miRNA cluster is also present in MSCs-derived exosomes, being formed by miR-17, miR-18a, miR-19a/b, miR-20a, and miR-90a, and being described as important modulators of neurite remodeling and neurogenesis, as well as stimulators of axonal growth and CNS recovery [119]. For instance, mimics, such as mimic-miR-124, are able to promote subventricular zone (SVZ) neurogenesis, which was shown after intracerebral administration in a 6-OHDA mice model of PD, and was also correlated with significant behavioral improvements [120]. In contrast, the mimic-miR-7 is also able to suppress NLRP3 and α-synuclein in the nigrostriatal pathway, thereby providing a potential therapeutic effect for PD. Regarding the antago-miRs, the antago-miR-155 may be relevant to PD therapy, since miR-155 plays a key role in the microglial cells activation in PD, leading to neuroinflammation. Finally, the overexpression of miR-126 leads to an impairment in the IGF-1 signaling, increasing DAn vulnerability to the PD neurotoxin, 6-OHDA. Notwithstanding, when using an antago-miR-126, the opposite occurs, resulting in neuroprotective effects induced by IGF-1 [121]. 

In summary, the development of an understanding of the molecular mechanisms regulated by miRNAs and the potential of MSCs-derived exosomes in how they impact PD brain homeostasis may allow the creation and development of important clinical gains to be translated to PD patients.

## 6. Conclusions and Future Perspectives

PD is a severe neurodegenerative disease that affects millions of people worldwide, and despite the advances in the PD research field, the molecular and cellular basis underlying this disease are still not fully understood. While important gains were achieved with the current pharmacological/surgical treatments in the quality of life of PD patients, they have failed to arrest PD progression and do not promote DAn protection/differentiation. Thus, a new approach that allows an understanding of the cellular and molecular mechanisms of PD to identify new therapeutical strategies and targets is necessary. Currently, MSC-secretome has been proposed as a promising therapeutic tool for several neurodegenerative diseases, like PD, given their ability to modulate DAn survival. Within it, MSCs-derived exosomes constitute, along with the protein fraction, an important tool and therapeutic option. Indeed, the exchange of genetic material, such as miRNA, through exosomes can promote neurogenesis, reduce neuroinflammation, as well as promote functional recovery in animal models. In fact, miRNAs have gained an important status in the PD research field not only due to its involvement in PD pathogenesis, but also as an opportune window to use as biomarkers or as potential therapeutic agents for PD treatment. Therefore, understanding the complexity of MSCs-derived exosomes, and how its miRNA content interacts with the molecular and cellular PD mechanisms is of great importance. Such an approach will not only allow the exploitation of potential pathways involved in the recovery/compensation mechanisms of the disease, but also in the development of multi-target-based strategies that could generate potential clinical benefits to be translated for PD patients.

## Figures and Tables

**Table 1:** Sporadic and genetic types of Parkinson’s Disease (PD).

**Sporadic PD**	
**Disruption of protein clearance pathways** **Accumulation of** **α** **-synuclein protein** **Mitochondrial dysfunction** **Excitotoxicity** **Oxidative stress** **Neuroinflammation**	
**Genetic PD**	
**SNCA**	Accumulation of α-synuclein protein aggregates.
**PRKN**	Decrease in DJ-1 and PARKIN proteins, which leads to mitochondria dysfunction when in oxidative stress conditions.
**PARK7**	
**UCHL-1**	No stabilization of ubiquitin monomers, which can lead to ubiquitin-proteasome system dysfunction.
**PINK1**	Reduction in PTEN induced putative kinase 1 activity, which can lead to mitochondria malfunction.
**PARK8**	Overexpression of LRRK2 that causes DAn loss, accompanied by the presence of LB.
